# Fluctuations in angle lambda with the pupil diameter and correlations with biometric values in a healthy population

**DOI:** 10.1111/opo.70006

**Published:** 2025-08-14

**Authors:** Maxence Rateaux, Alienor Vienne‐Jumeau, Dominique Bremond‐Gignac, Matthieu P. Robert

**Affiliations:** ^1^ Ophthalmology Department University Hospital Necker‐Enfants Malades, APHP Paris France; ^2^ Centre Borelli ENS Paris‐Saclay, CNRS, INSERM, SSA, Paris Cité University Paris France; ^3^ CHNO des Quinze‐Vingts INSERM‐DGOS CIC Paris France; ^4^ INSERM UMRS 1138, T17, Centre des Cordeliers Centre de Recherche des Cordeliers, Sorbonne Paris Cité University Paris France

**Keywords:** angle lambda, biometry, photograph, pupil

## Abstract

**Purpose:**

Angle lambda (*λ*) is defined as the angle between the line of sight and the pupillary axis at the entrance pupil. We previously developed a child‐friendly and portable method to measure this angle in daily practice. In a given population, angle *λ* fluctuates according to age or refractive error. As changes in the pupil diameter induce changes in the location of the pupil centre, it was hypothesised that a given subject will exhibit several angles *λ*, varying with the luminance level. The study aimed to investigate correlations between angle *λ* and biometric values and to analyse the effects of pupil dilation and entrance pupil location on angle *λ*.

**Method:**

The study was performed on 70 right eyes from 70 participants (58 women, 12 men; mean age 22.91 ± 2.57 years). Angle *λ* was assessed under photopic and scotopic luminance conditions. Angle *λ* was also measured under standard luminance conditions in a subgroup of 15 eyes. Axial length, anterior chamber depth, chord *μ* (coaxial corneal light reflex position) and the optic disc to fovea distance (OFD) were also measured. The pupil centre offset was quantified by digital analysis.

**Results:**

Mean photopic and scotopic angle *λ* values were +2.81 ± 2.34° and +3.30 ± 2.59°, respectively. A negative correlation was found between axial length, anterior chamber depth or OFD and both photopic and scotopic angle *λ*. A positive correlation was found between spherical equivalent or chord *μ* and angle *λ*. The mean pupil offset was significantly higher under photopic than scotopic conditions and was negatively correlated with angle *λ* for both luminance levels.

**Conclusion:**

This study confirms that angle *λ* is correlated with biometric values. Furthermore, fluctuation of pupil diameter induces variations in angle *λ*. Thus, a given subject exhibits several angles *λ* according to the luminance level.

**Key points**

- Angle lambda is positively correlated with the spherical equivalent refractive error and negatively correlated with axial length, anterior chamber depth and optic disc to fovea distance.
- Angle lambda fluctuates over time due to the nasal shift of the pupil during miosis, and its value is significantly lower in photopic conditions.
- An individual exhibits several values of angle lambda, varying with luminance; however, these fluctuations have little clinical relevance in strabismus management.

## INTRODUCTION

**FIGURE 1:**
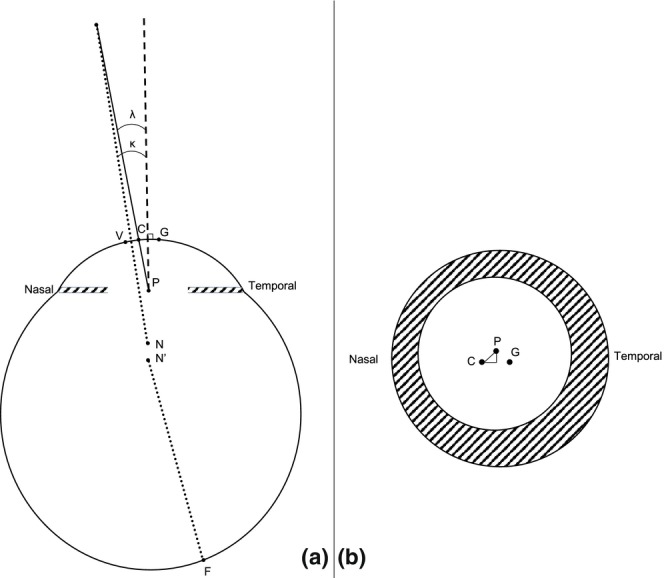
Model eye. (a) Horizontal view. (b) Frontal view. V: Corneal vertex; C: Corneal light reflex; G: Geometric centre of the cornea; P: Centre of the entrance pupil; F: Fovea; N and N′: Theoretical position of two main nodal points; Dotted line: Visual axis; Dashed line: Pupillary axis; Solid line: Line of sight; *λ*: Angle lambda; *κ*: Angle kappa. The right triangle in (b) represents the mathematical construction of chord μ (CP).

The anatomical and dynamic description of the eyeball provided by physiological optics relies on several angles formed by the intersection of a number of axes.^1^ Some definitions lack consensus, and variations are encountered in the literature.^2^ Here, angle alpha (*α*) is defined as the angle formed by the optical axis (a line connecting the nodal point and the apex of the cornea) and the visual axis (shown in Figure 1 as a broken line between the fixation point and the fovea, passing through the nodal point). Angle kappa (*κ*) is defined as the angle between the visual axis and the pupillary axis (line passing through the centre of the entrance pupil, orthogonal to the cornea). Moreover, as the human eye is not a centred optical system, the nodal point does not have an anatomical location.^3^, ^4^ Therefore, these angles cannot be used clinically. This implies that angle lambda (*λ*) is the only angle accessible for clinical examination. It is formed between the pupillary axis and the line of sight (a line connecting the centre of the entrance pupil and the fixation point) (Figure 1). In the literature, inconsistent definitions have been employed for angles *κ* and *λ*.^1^, ^2^ It is generally accepted, however, that the values of angles *κ* and *λ* would be very close, especially during distance fixation.^1^

In clinical practice, corneal topographers and synoptophores can provide an estimation of angle *λ* –although it is often mistakenly designated as angle *κ*.^5^, ^6^, ^7^ Its evaluation is crucial in refractive surgery for ensuring the proper centration of the optical zone; it can also be utilised in cataract surgery for determining the optimal intraocular lens position.^8^, ^9^ Moreover, it plays an essential role in strabismus management, as an out‐of‐range angle *λ* can significantly affect the visual appearance of the ocular deviation.^10^, ^11^, ^12^ We recently developed an easy, reproducible and cost‐effective method for assessing angle *λ*. This method relies on monocular flash photographs of both eyes, employing a ring flash around the camera lens and a fixation target positioned at its centre.^13^

In a healthy population, angle *λ* typically ranges between 1° and 5°; it has been shown to decrease with age and to vary with axial length and spherical equivalent (SE) refractive error.^6^, ^14^ Various disorders, such as albinism, congenital aniridia and retinal folds, can alter the value of angle *λ*, usually resulting in a high positive value.^15^, ^16^ In a given individual, it is not clear whether angle *λ* is stable or if it varies with physiological factors. It is known that the pupil diameter fluctuates over time due to intrinsic factors, such as age and emotion, as well as extrinsic factors, particularly ambient luminance. If such dilation or miosis were concentric, then it would have no consequence on angle *λ*. However, the pupil usually shifts nasally during miosis.^17^, ^18^ This fluctuation causes displacement of the location of the entrance pupil centre (LEPC) relative to the geometrical centre of the cornea, and consequently, of the pupillary axis. In this context, there could theoretically be multiple angles *λ* for an individual, as previously suggested by Gullstrand.^1^ Our hypothesis is that miosis will reduce the angle between the pupillary axis and the line of sight, thereby decreasing angle *λ*.

The objectives of this study are firstly to replicate previously published results showing a significant correlation between angle *λ* and biometric parameters such as axial length, anterior chamber depth and the distance from the optic disc to the fovea in a healthy population; and secondly to investigate the influence of the pupil diameter on angle *λ*.

## METHODS

The study was conducted at the ophthalmology department of Necker‐Enfants Malades University Hospital, Assistance Publique – Hôpitaux de Paris (AP‐HP) and received approval from the Ethical Committee of Île‐de‐France (Study IDRCB 2020‐A03370‐39).

Seventy healthy subjects (70 right eyes) aged between 18 and 31 years were recruited. Exclusion criteria included the presence of any ocular disorder, strabismus or reduced visual acuity. To account for potential inter‐eye dependence and to avoid hysteresis effects related to the measurement being performed first on the right eye, only data from the right eyes were used.

The study comprised three parts. Part one was to confirm that the subjects met the eligibility criteria. It involved measuring visual acuity (for both far and near vision, using decimal [nidek.fr)] and Parinaud charts and converted into logMAR), assessing stereoacuity [TNO test,(lameris‐group.nl) and conducting a cover‐test to confirm the absence of strabismus. In part two, a multimodal imaging sequence was performed to assess biometric values. The optic disc to fovea distance (OFD) was measured in degrees using the Canon CR‐2 plus AF retinal camera (fr.medical.canon), and image magnification was corrected using the Littmann‐Bennett formula and the axial length method.^19^, ^20^ The OFD was also measured in mm using the OPTOS Daytona ultra‐wide field retinophotographer (WFRP) (optos.fr). Corneal topography was performed using the Oculus Pentacam (pentacam.com) to measure the corneal diameter and evaluate chord *μ* using the formula: $\text{Chord}\;\mu =\sqrt{x_{\text{pupil}\_\text{centre}}^{2}+y_{\text{pupil}\_\text{centre}}^{2}}$ (*x* and *y* correspond to the horizontal and vertical position of the pupil centre from the corneal vertex [in mm], respectively).^2^ Ocular biometry was conducted using the Zeiss IOL Master 500 (zeiss.com/meditec) to measure the axial length (AL) and anterior chamber depth (ACD). Part three involved assessing angle *λ*. Two monocular photographs of the right eye were taken using a Nikon D5300 camera equipped with a ring flash around the lens and a fixation target placed at the centre to achieve a perfectly coaxial sighted corneal light reflex. The first sequence was performed under photopic conditions (illuminance = 1297 lux (±3% for device measurement)); a monocular picture with maximum miosis was captured using an extended flashlight. The second sequence was conducted under scotopic conditions (illuminance <0.1 lux (±3%)). The participant was instructed to maintain straight gaze on the target. Then, the light was turned off to achieve full mydriasis and a photograph taken. In a subgroup of 15 right eyes from 15 subjects, photographs were also taken under standard photopic conditions (which corresponded to moderate levels of illuminance in the examination room (illuminance = 138 lux (±3%))), to obtain values of angle *λ* (termed standard *λ*) under ‘standard’ clinical conditions.

The calculation of angle *λ* used the monocular photograph‐based method as described previously.^13^ Picture analysis was conducted using the free image editor GIMP software (v2.10.14, gimp.org). This analysis allowed the calculation of angle *λ* and LEPC in pixels, which were then converted to degrees and millimetres, respectively.

Statistical analysis was conducted using R Studio (Version 2024) (cran.r‐project.org). The normal distribution of all biometric values and angles was assessed using the Kolmogorov–Smirnov test. Paired analysis of variance (ANOVA) tests were used to compare the mean angle *λ* under photopic, scotopic and standard conditions. Pearson correlation coefficients were computed to assess the linear relationship between photopic and scotopic angle *λ* and the following ocular parameters: SE, chord length, AL, ACD and both photopic and scotopic LEPC and OFD distances. Univariate and multivariate linear regression analyses were performed using the lm function. Model comparisons were conducted using ANOVA with nested *F*‐tests to assess the contribution of each variable. The significance threshold was set at *α* = 0.05. Adjusted *R* ^2^ values were used to evaluate the model fit.

## RESULTS

Seventy subjects (70 eyes from 58 women and 12 men) were enrolled, with an age range of 18.4–30.2 years (mean = 22.91 ± 2.57 years). Biometric parameters are detailed in Table 1. A normal distribution was confirmed for all biometric values and angles (*p* > 0.05).

**TABLE 1:** Distribution of biometric parameters for the right eye.

	Mean ± SD	Med (min–max)	95% CI
SE (in D)	−0.78 ± 1.91	−0.44 (−6.75 to +6.00)	−1.23 to −0.33
Chord *μ*	0.22 ± 0.125	0.20 (0.01–0.78)	0.19 to 0.25
AL (in mm)	23.76 ± 1.14	23.75 (21.32–26.73)	23.50 to 24.03
ACD (in mm)	3.57 ± 0.25	3.6 (2.86–4.12)	3.51 to 3.63
CD (in mm)	11.87 ± 0.46	11.8 (11.0–13.1)	11.77 to 11.98
OFD measured with ultra‐wide field RP (in mm)	4.64 ± 0.26	4.65 (4.2–5.4)	4.58 to 4.70
OFD measured with conventional RP (in°)	15.92 ± 1.12	15.98 (13.75–18.71)	15.66 to 16.18

Abbreviations: ACD, Anterior chamber depth; AL, Axial length; CD, Corneal diameter; CI, confidence intervals; D, Dioptre; OFD, Optic‐disc to fovea distance; RP, retinal photographs; SE, Spherical equivalent.

### Distribution of angle *λ*

**FIGURE 2:**
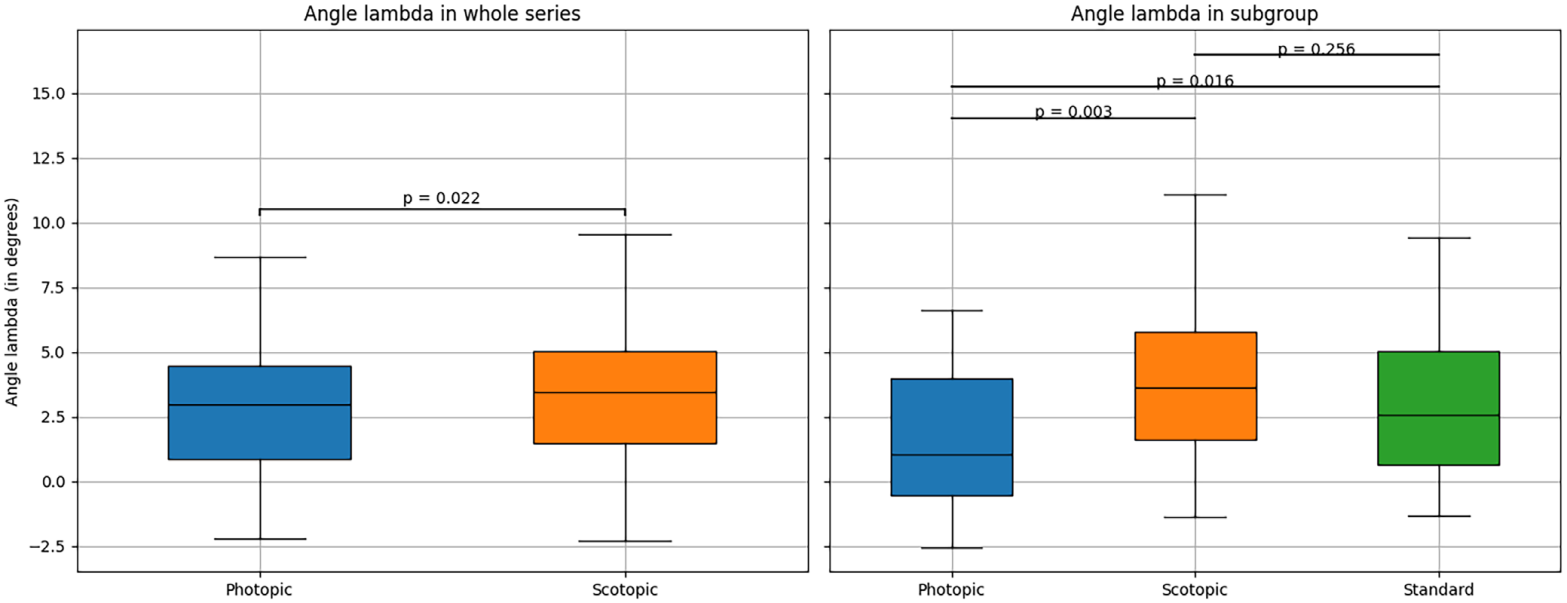
Distribution of angle *λ* under photopic and scotopic conditions for all subjects (whole series, left) and in the subgroup (*N* = 15) where measurements were also recorded under standard luminance (right).

The distribution of angle *λ* under different luminance conditions is illustrated in Figure 2. The mean photopic and scotopic angle *λ* was +2.81° ± 2.34° and +3.30° ± 2.59°, respectively (*p* = 0.02). Note that negative and positive values indicate a temporal or nasal location of the corneal reflex, respectively. The average of the photopic and scotopic values was +3.06° ± 2.26°. The standard angle *λ* measurements recorded for the subgroup ranged from −1.31° to +9.41°, with a mean value of +3.14° (median = +2.59; SD = 3.04). There was no significant difference between the standard angle *λ* and that obtained under scotopic conditions (*p* = 0.26), while significant differences were found between the mean photopic angle and both the standard and scotopic angles (*p* = 0.02 and 0.003, respectively). Standard angle *λ* was positively correlated with both the photopic and scotopic angles (*r* = 0.78; *p* = 0.0006; *r* = 0.67; *p* = 0.006, respectively).

### Biometrical values and correlation with angles

Correlations between the photopic and scotopic angles and biometric values are detailed in Table 2. AL range was positively correlated with ACD (*r* = 0.74; *p* < 0.0001). Significant negative correlations were observed between AL and both the photopic and scotopic angles. Moreover, a significant negative correlation was found between ACD and photopic angle *λ*. No significant correlation was seen between angle *λ* and the corneal diameter.

**TABLE 2:** Correlation between photopic and scotopic angles *λ* and biometric values for the right eye.

	Photopic angle *λ*		Scotopic angle *λ*	
	Pearson	*p*‐value	Pearson	*p*‐value
SE (in D)	**0.46**	**<0.0001**	**0.31**	**0.01**
Chord *μ*	**0.65**	**<0.0001**	**0.59**	**<0.0001**
AL (in mm)	**−0.39**	**0.002**	**−0.25**	**0.03**
ACD (in mm)	**−0.35**	**0.003**	−0.18	0.14
CD (in mm)	−0.12	0.30	−0.01	0.93
OFD measured with ultra‐wide field RP (in mm)	−0.06	0.63	−0.09	0.48
OFD measured with conventional RP (in°)	**−0.40**	**0.0005**	**−0.38**	**0.002**

Abbreviations: ACD, Anterior chamber depth; AL, Axial length; CD, Corneal diameter; D, Dioptre; OFD, Optic disc to fovea distance; RP, retinal photography; SE, Spherical equivalent. Bold values indicates significant results (*p* < 0.05).

The OFD measured with conventional retinal photographs was positively correlated with the distance measured using the OPTOS WFRP (*R* ^2^ = 0.18; *p* = 0.0002). A negative correlation was found between the OFD measured by retinal photography and both photopic and scotopic angles *λ* (*r* = −0.40; *p* = 0.0005 and *r* = −0.38; *p* = 0.002, respectively).

### Variation of angles with light conditions

Fluctuations of angle *λ* were assessed by the difference in values of angle *λ* under scotopic and photopic conditions; the mean of fluctuations was +0.49° ± 1.99 (min = −4.50°; max = +6.50°). The average pupil diameter under photopic and scotopic conditions was 3.08 mm ± 0.65 mm and 5.57 mm ± 0.81 mm, respectively. LEPC values ranged from 0 to +0.441 mm under photopic conditions (mean = 0.193 ± 0.098) and from −0.156 mm to +0.351 mm under scotopic conditions (mean = 0.131 ± 0.10).

The scotopic and photopic LEPC values were positively correlated with one another (*r* = 0.45; *p* < 0.0001). The mean photopic LEPC value was significantly higher than the scotopic LEPC (*p* < 0.0001). LEPC was negatively correlated with angle *λ* for the photopic condition (*r* = −0.37; *p* = 0.002). A negative correlation was also found under the scotopic condition (*r* = −0.22, *p* = 0.07). The mean LEPC (average of photopic and scotopic LEPC) was 0.16 ± 0.08 mm. A significant correlation was found between mean LEPC and AL (*r* = −0.28; *p* = 0.02). Correlations between LEPC and the biometric findings are detailed in Table 3.

**TABLE 3:** Correlations between the location of the entrance pupil centre and biometric values for the right eye.

	Photopic LEPC		Scotopic LEPC		Mean LEPC	
	Pearson	*p*‐value	Pearson	*p*‐value	Pearson	*p*‐value
SE (in D)	0.03	0.80	**0.34**	**0.004**	0.22	0.06
AL (in mm)	−0.20	0.09	**−0.27**	**0.02**	**−0.28**	**0.02**
ACD (in mm)	−0.11	0.37	−0.23	0.05	−0.20	0.09
CD (in mm)	−0.22	0.07	−0.10	0.42	−0.18	0.13
OFD measured with ultra‐wide field RP (in mm)	0.13	0.30	**0.31**	**0.009**	**0.26**	**0.03**
OFD measured with conventional RP (in°)	−0.04	0.77	0.07	0.56	0.03	0.85

Abbreviations: ACD, Anterior chamber depth; AL, Axial length; CD, Corneal diameter; D, Dioptre; LEPC, Location of the entrance pupil centre; OFD, Optic disc to fovea distance; RP, retinal photography; SE, Spherical equivalent. Bold values indicates significants results (*p* < 0.05).

### Multiple linear regression

**FIGURE 3:**
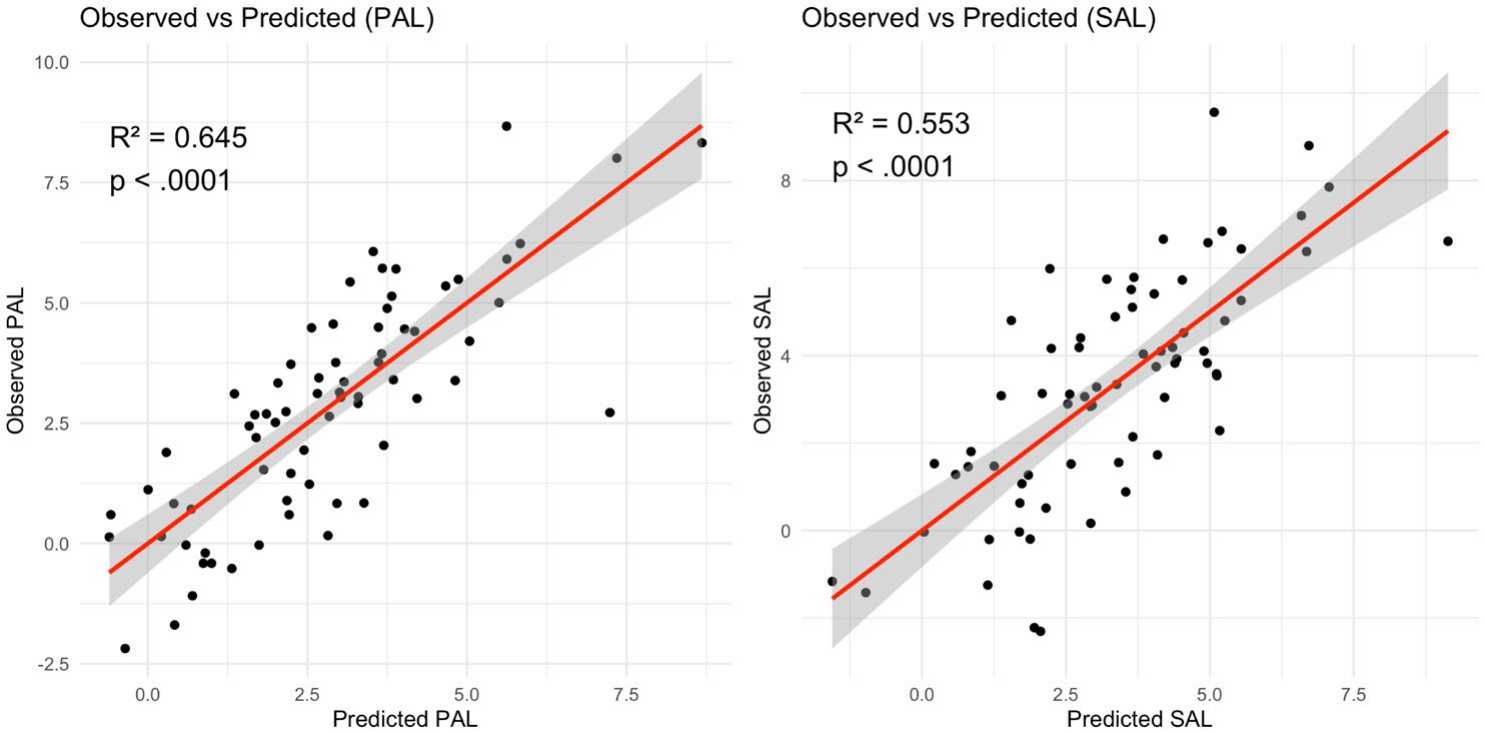
Actual versus predicted values of angle *λ* from the multilinear regression model, under photopic (a) and scotopic (b) conditions. The plot illustrates the model's goodness of fit, with data points clustering around the identity line (*y* = *x*) indicating accurate predictions. The shaded area corresponds to the 95% confidence interval of the linear regression line. PAL, photopic angle lambda; SAL, scotopic angle lambda.

Table 4 shows the results from multivariable linear regression models. In the analysis, chord *μ* and photopic LEPC were significantly correlated with photopic angle *λ*, while SE showed a trend‐level association (*p* = 0.05). The overall model including chord *μ* and photopic LEPC was significant (adjusted *R* ^2^ = 0.42; *p* < 0.0001). Adding SE to a model with chord *μ* and photopic LEPC significantly improved the model performance (*F* = 23.06, *p* < 0.0001). The resulting regression equation was: photopic angle *λ* = 1.978 + 0.473 × SE + 9.71 × chord *μ* + −4.77 × photopic LEPC (adjusted *R* ^2^ = 0.56; *p* < 0.0001). For scotopic angle *λ*, chord *μ*, photopic LEPC and scotopic LEPC were significantly associated (adjusted *R* ^2^ = 0.55; *p* < 0.0001). Adding SE significantly improved the model fit (*F* = 14.76, *p* = 0.0003). The final regression model was: scotopic angle *λ* = 0.823 + 0.478 × SE + 12.93 × chord *μ* + 7.6 × photopic LEPC + −11.404 × scotopic LEPC (adjusted *R* ^2^ = 0.38; *p* = 0.002). Figure 3 shows the observed versus predicted values under both luminance conditions.

**TABLE 4:** Multiple linear regression to identify contributing factors that influenced angle lambda under both luminance conditions.

	Photopic angle *λ*		Scotopic angle *λ*	
	Estimate	*p*‐value	Estimate	*p*‐value
SE (in D)	0.33	0.05	0.34	0.10
Chord *μ*	9.70	**<0.0001**	12.45	**<0.0001**
AL (in mm)	−0.11	0.81	0.27	0.63
ACD (in mm)	−1.21	0.29	−0.10	0.95
CD (in mm)	−0.68	0.19	0.21	0.74
OFD measured with ultra‐wide field RP (in mm)	−1.26	0.35	1.57	0.35
OFD measured with conventional RP (in°)	−0.09	0.82	−0.68	0.15
Photopic LEPC (in mm)	−5.55	**0.03**	6.83	**0.03**
Scotopic LEPC (in mm)	−0.21	0.93	−10.05	**0.001**

Abbreviations: ACD, Anterior chamber depth; AL, Axial length; CD, Corneal diameter; D, Dioptre; LEPC, Location of the entrance pupil centre; OFD, Optic disc to fovea distance; RP, retinal photography; SE, Spherical equivalent. Bold values indicates significant results (*p* < 0.05).

## DISCUSSION

This study aimed to examine correlations between angle *λ* and biometric parameters and to assess variations of this angle with differences in pupil diameter. Significant correlations were observed between angle *λ* (measured under both photopic and scotopic conditions) and SE, AL and OFD assessed by conventional retinal photography. Furthermore, angle *λ* was significantly smaller under photopic than scotopic conditions.

The mean angle *λ* in healthy subjects was +3.06° ± 2.26°. This result closely aligns with the findings of Yeo et al. (+3.19° ± 1.15°), who employed a mathematical model based on ACD measurements from ultrasound biomicroscopy (UBM).^5^ Conversely, Gharaee et al., using the Bausch and Lomb Orbscan II (bausch.com), reported a mean angle *λ* of +4.96° ± 1.86°.^14^ These higher values could be attributed to methodological differences. The Orbscan II tends to overestimate angle *λ*, likely due to ACD underestimation compared with UBM or the IOLMaster.^5^, ^21^, ^22^

The positive correlation observed between SE and angle *λ* under both luminances echoes previous findings. Both Reinstein et al. and Gharieb et al. noted significantly higher angle *λ* values in hyperopic individuals compared with myopes.^23^, ^24^ Additionally, the positive correlation between AL and OFD, when measured using RP, aligns with the findings of Jonas et al.^25^

Classically, measurements of retinal distances are performed using conventional retinal photography, with the Littman coefficient for correcting image magnification. Here, fundus distances were also measured using the OPTOS WFRP. Although distances measured using these two methods were positively correlated, the *R* ^2^ value was unexpectedly low (0.18); therefore, we chose not to use the OPTOS WFRP method for the multiple linear regression analyses, considering that the photographic method can be considered as the reference, having been used extensively in previous work.^20^

A significant negative correlation was found between OFD measured with retinal photography and angle *λ* under both luminance conditions. This finding was not expected. Logically, temporal displacement of the OFD should induce a rotation of the line of sight to maintain central fixation. This should result in a nasal shift of the line of sight relative to the pupillary axis, and therefore an increased angle *λ*. This hypothesis is supported by findings in pathological conditions, such as albinism, where the fovea is displaced temporally, the OFD is increased and angle *λ* is abnormally positive.^26^, ^27^ However, in a healthy population, the range of OFD is relatively narrow, which limits the ability to detect a significant relation between OFD and angle *λ*. Additionally, the AL and the OFD are positively correlated with one another, while the AL and angle *λ* are negatively correlated. Consequently, we hypothesise that these opposite correlations may cancel each other out and mask the expected inverse correlation between OFD and angle *λ*.

Moreover, we found that the LEPC was displaced slightly nasally relative to the *geometrical centre* of the cornea under both luminance conditions. This finding is consistent with the conclusions of Yang et al.^18^ However, other studies have reported a temporal ‘pupil offset’.^23^, ^28^ This discrepancy stems from the anatomical structure used as a reference; the LEPC is defined as the position of the centre of the entrance pupil seen through the cornea, compared with the position of the *geometrical centre* of the cornea, while the ‘pupil offset’ designates the distance between the pupillary axis and the *corneal vertex*. The *corneal vertex* is located nasally from the *geometrical centre* of the cornea, and the pupillary axis usually falls in between these two points (Figure 1).^23^, ^28^, ^29^ This difference is exemplified by Yang et al.'s findings, indicating a nasal position of both the pupil centre and the first Purkinje image compared with the geometrical centre.^18^

As the pupil diameter changes, there is a consistent displacement of the LEPC, induced by the nasal shift of the pupil during miosis.^18^, ^28^, ^30^ In the present work, the mean decentration under photopic conditions exceeded that found under scotopic conditions (*p* < 0.0001), logically resulting from this pupil displacement. Finally, each participant exhibited a specific angle *λ*, which fluctuates over time according to the pupil diameter, decreasing with miosis.

Multiple linear regression highlighted the multifactorial outcome of angle *λ*, with the pupil position having a higher impact than other biometric parameters. Gharieb et al. found a significant impact of SE, AL, ACD and pupil radius on angle *λ*. Given the strong correlation between SE, AL and ACD, it is difficult to formulate hypotheses regarding the causality links between these biometric parameters and angle *λ*.

Chang and Waring proposed the use of chord *μ* as a new clinical reference in refractive surgery.^2^ Based on the two‐dimensional relation between the line of sight and the subject‐fixating, coaxially sighted, corneal light reflex at the corneal surface, this marker (which is not an angle) disregards the three‐dimensional characteristics of the anterior chamber, while taking into account the pupil diameter. Then, only topographic or photographic data are needed. A positive correlation was found between chord *μ* and both photopic (*r* = 0.65) and scotopic angle *λ* (*r* = 0.59). While this recently described anatomical landmark is relevant for refractive surgery, its clinical pertinence is questionable in the context of strabismus management. Indeed, ocular deviations depend on anatomical parameters such as pupillary distance and external parameters such as fixation distance. Therefore, adopting three‐dimensional landmarks and angular measurements, rather than relying solely on two‐dimensional components, appears more suitable and precise in the assessment of strabismus.

Currently, there is no angle which can be used clinically that is independent of the pupil diameter. Subjective methods for quantification of the ocular deviation, such as the Krismsky test, require identification of the position of the corneal light reflex, relative to the pupillary axis, which corresponds with angle *λ*.^31^ However, in the subgroup of 15 eyes, angles *λ* measured under photopic, scotopic and standard luminance conditions were strongly positively correlated, suggesting that fluctuations in the angle, resulting from changes in pupil diameter, likely have little consequence in clinical practice. Nevertheless, in order to ensure reproducible measurements, it may be advisable to record the parameter under fixed luminance conditions.

## CONCLUSION

Angle *λ*, which is formed by the pupillary axis and the line of sight, is clinically accessible, unlike angles *α* or *κ*. This study confirms that in a healthy population, the value of angle *λ* is significantly correlated with biometric values, such as AL, ACD and the OFD. Furthermore, it has been demonstrated that angle *λ* fluctuates over time for an individual. These fluctuations are dependent on the pupil diameter, as the pupil and its centre shift nasally during miosis. However, the values of angle *λ* under varying illumination conditions are correlated, suggesting that ‘standard’ illumination measurements can be used reliably in daily practice.

## AUTHOR CONTRIBUTIONS

**Maxence Rateaux:** Conceptualization, formal analysis, investigation, methodology, project acquisition, resources, validation, visualization, writing—original draft preparation, writing—review and editing. **Alienor Vienne‐Jumeau:** Formal analysis, software, visualization, writing—original draft preparation, writing—review and editing. **Dominique Bremond‐Gignac:** Resources, supervision, visualization, writing—original draft preparation, writing—review and editing. **Matthieu P. Robert:** Resources, supervision, validation, visualization, writing—original draft preparation, writing—review and editing.

## FUNDING INFORMATION

No funding.

## CONFLICT OF INTEREST STATEMENT

The authors report no conflicts of interest and have no proprietary interest in any of the materials mentioned in this article.
